# Corneal myofibroblasts inhibit regenerating nerves during wound healing

**DOI:** 10.1038/s41598-018-30964-y

**Published:** 2018-08-28

**Authors:** Kye-Im Jeon, Holly B. Hindman, Tracy Bubel, Thurma McDaniel, Margaret DeMagistris, Christine Callan, Krystel R. Huxlin

**Affiliations:** Flaum Eye Institute, University of Rochester, Rochester, NY USA

## Abstract

Abnormal nerve regeneration often follows corneal injury, predisposing patients to pain, dry eye and vision loss. Yet, we lack a mechanistic understanding of this process. A key event in corneal wounds is the differentiation of keratocytes into fibroblasts and scar-forming myofibroblasts. Here, we show for the first time that regenerating nerves avoid corneal regions populated by myofibroblasts *in vivo*. Recreating this interaction *in vitro*, we find neurite outgrowth delayed when myofibroblasts but not fibroblasts, are co-cultured with sensory neurons. After neurites elongated sufficiently, contact inhibition was observed with myofibroblasts, but not fibroblasts. Reduced neurite outgrowth *in vitro* appeared mediated by transforming growth factor beta 1 (TGF-β1) secreted by myofibroblasts, which increased phosphorylation of collapsin response mediating protein 2 (CRMP2) in neurons. The significance of this mechanism was further tested by applying Mitomycin C after photorefractive keratectomy to decrease myofibroblast differentiation. This generated earlier repopulation of the ablation zone by intra-epithelial and sub-basal nerves. Our findings suggest that attaining proper, rapid corneal nerve regeneration after injury may require blocking myofibroblast differentiation and/or TGF-β during wound healing. They also highlight hitherto undefined myofibroblast-neuron signaling processes capable of restricting neurite outgrowth in the cornea and other tissues where scars and nerves co-exist.

## Introduction

The cornea is the most densely innervated peripheral tissue in the human body^1^ and its exquisite sensory abilities are designed to protect the eye from outside elements. The peripheral nerves in the cornea are predominantly sensory and nociceptive, coding discomfort and pain in response to mechanical stimulation, temperature change and/or chemical stimulation. In addition, they control the blink reflex, tear production and tear secretion (reviewed in^2–5^). Disease, infection and surgery can all damage corneal nerves, with long-term consequences among a wide range of patient populations^5^. At worse, blindness ensues^6^. When nerve damage is less severe, pain^7,8^ and dry eye^9^ appear to be the main symptoms, decreasing quality of life for those affected. Even after Laser *in situ* keratomileusis (LASIK), the most popular form of laser refractive surgery today, it appears that corneal nerve density, morphology and function never completely return to normal

Despite its clinical relevance, our knowledge about corneal nerve regeneration following injury and diseases such as neurotrophic keratitis remains sub-optimal^10^. Many studies have demonstrated interplay between immune cells and corneal nerves (e.g.^11,12^), as well as between nerves and the corneal epithelium, including stem cells^13–16^. Experimentally, in addition to healing the epithelium, the most common approach to enhance corneal nerve regeneration is to apply growth factors such as NGF and VEGF to the ocular surface^17,18^. However, this approach ignores the potential contribution of the stromal environment to nerve regeneration, and with one exception^19^, the interaction between nerves and stromal keratocytes, fibroblasts or myofibroblasts remains unexplored. This is an important gap to fill given that keratocytes occupy and synthesize the bulk of the cornea’s volume (the stroma), are critical for maintaining the cornea’s unique optical and structural properties, and transform into fibroblasts and scar-forming myofibroblasts during wound healing^20–22^.

Mammalian corneal nerves arise from trigeminal ganglion cells, whose axons enter the cornea at the limbus of the eye. They course in thick cords through the anterior stroma until they ramify to form the sub-basal plexus, from which intra-epithelial nerves derive^4,23,24^. Thus, a large proportion of corneal nerves reside in the stroma, where their main cell-to-cell interaction is with keratocytes in the healthy, resting state, and with fibroblasts and myofibroblasts following injury or surgery. A recent study reported that serum-free medium conditioned by cultured, human corneal fibroblasts (but not keratocytes) promoted neurite outgrowth in cultured chick dorsal root ganglion cells in a dose-dependent manner^19^. Here, we asked how fibroblasts compare to myofibroblasts in terms of their effect on neurite outgrowth. We first answered this question by systematically characterizing nerve regeneration at different time-points in corneas from a large animal (domestic cat) following corneal wounds created with photorefractive keratectomy (PRK). This widely used clinical technique was employed here to reproducibly generate a “controlled” injury that destroyed nerve fibers in the central epithelium, sub-basal layer and stroma. Because the wound healing reaction that follows PRK in our animal model reliably includes large, persistent areas of differentiated myofibroblasts^20–22,25^, we were also able to examine the interaction between regenerating corneal nerves and these zones. To interrogate key molecular mechanisms underlying these interactions, we then co-cultured cat corneal fibroblasts/myofibroblasts with ND7/23 cells, which are created by fusing N18Tg2 mouse neuroblastoma cells and rat dorsal root ganglion cells^26^. ND7/23 cells have sensory neuron-like properties: they differentiate after inhibition of mitosis and extend neurites in the presence of nerve growth factor^27,28^, similar to those produced by dorsal root ganglion cells *in vitro*^26^.

A potentially important group of signaling molecules involved in the control of sensory neurite outgrowth belongs to the family of collapsin response mediator proteins (CRMPs). CRMPs were originally identified as mediators of semaphorin 3 A signaling^29^ and consist of five homologous, cytosolic, tubulin-binding proteins (CRMP1-CRMP5)^30^, which are phosphorylated by various kinases that regulate their activity^31,32^. CRMPs are most highly expressed during the neurogenic period of brain development, but also in sprouting central and peripheral nerve fibers after injury^33–36^. CRMP2, the first identified CRMP family member, has long been recognized as a critical regulator of axonal guidance and neuronal polarity *in vitro*^37^. Its activity is precisely regulated by phosphorylation status^38,39^: unphosphorylated CRMP2 promotes tubulin assembly and growth cone extension, whereas phosphorylation reduces CRMP2’s ability to interact with tubulin, inhibiting neurite extension^39^. Here we describe for the first time that phosphorylation of CRMP2 in cultured sensory neurons is modulated by transforming growth factor beta 1 (TGF-β1), and we provide evidence for a potentially key role of TGF-β1and p-CRMP2 in mediating the inhibitory effects of corneal myofibroblasts on regenerating nerves. Finally, in a key test of our premise that stromal cells are indeed critical determinants of corneal re-innervation after injury, we partially blocked myofibroblast differentiation post-PRK *in vivo* using Mitomycin C^40–43^, which significantly improved the rate at which nerves repopulated the ablation zone.

## Results

### Nerve distribution in intact corneas

**Figure 1:**
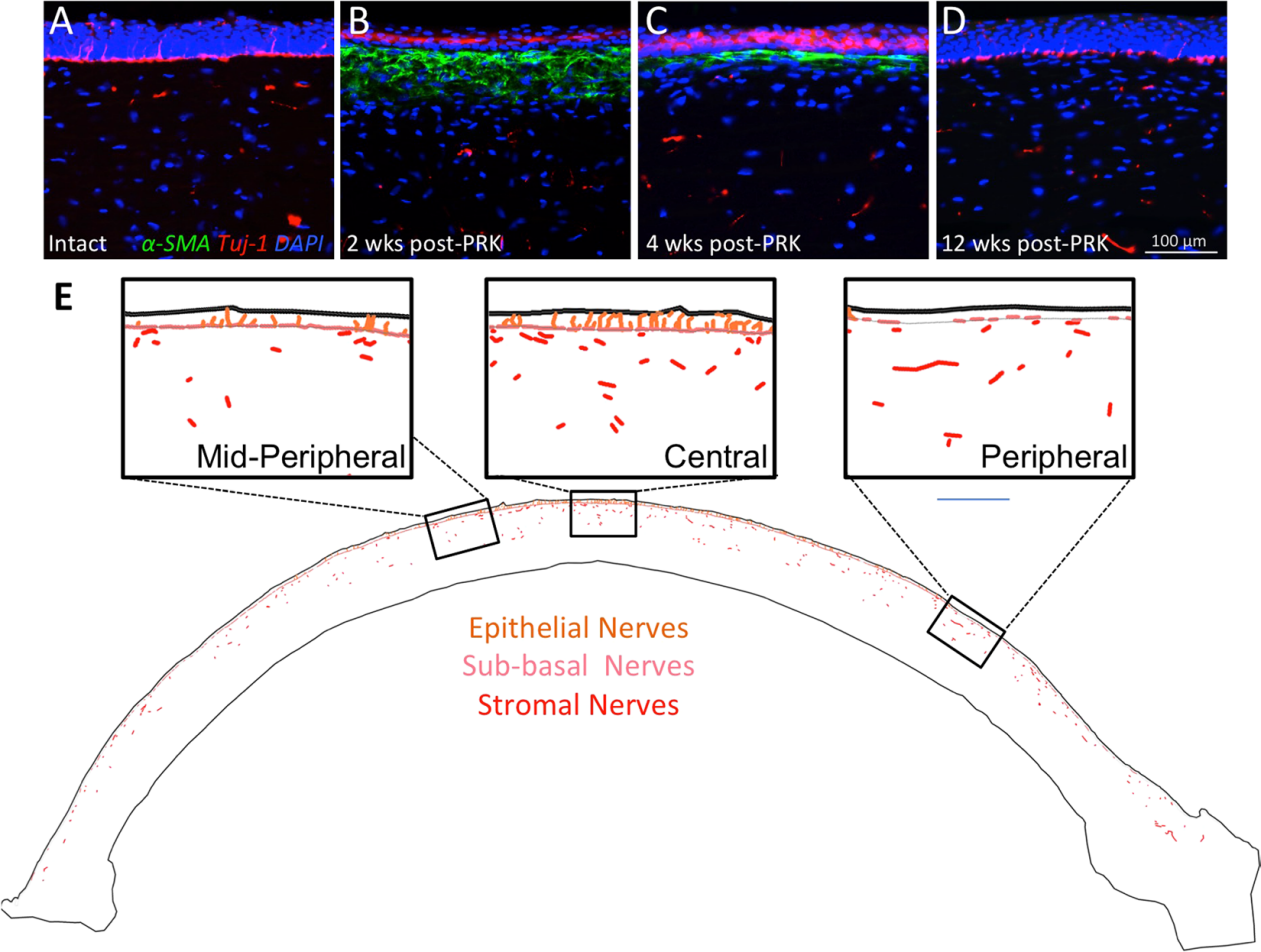
Immunohistochemical staining and analysis of feline corneas. (**A**) Photograph of normal, unoperated central cat corneal cross-section reacted for Tuj-1 (red fluorescence), α-SMA (green fluorescence) and counter-stained with DAPI (blue fluorescence). Note thick cords of Tuj-1 positive corneal nerves in the anterior stroma, the almost continuous sub-basal nerves right under the epithelium and the thin nerve endings visible between epithelial cells. (**B**) Photograph of the central cornea of a cat 2 weeks after PRK stained identically as in A. Note the distinct zone of positive α-SMA staining which lacks Tuj-1 positive nerves, the thinner but more densely distributed stromal nerves under that zone, and the Tuj-1 positive epithelium above that zone, which is devoid of intra-epithelial nerves. Note also the lack of sub-basal nerves. (**C**) Photograph of the central cornea of a cat 4 weeks after PRK, stained identically as in A and B. Note the thinner α-SMA positive zone, which remains devoid of nerves, in spite of their presence below it. The epithelium has increased in thickness, but also remains devoid of nerves. (**D**) Photograph of the central cornea in a cat 12 weeks after PRK showing a complete lack of α-SMA positive staining. Thicker trunks of Tuj-1 positive nerves are re-appearing in the stroma, as are intra-epithelial and sub-basal nerves. Scale bar = 100 µm for A-D. High-resolution monochrome views of Tuj-1 staining in (**A–D**) are presented in Fig. S1. (**E**) Tracing of an entire cat corneal cross section (unoperated cat) performed in Neurolucida, and illustrating with differential color-coding, the different compartments in which corneal nerve densities were analyzed. Insets show higher power views of the central, mid-peripheral and peripheral regions of the cornea.

**Figure 2:**
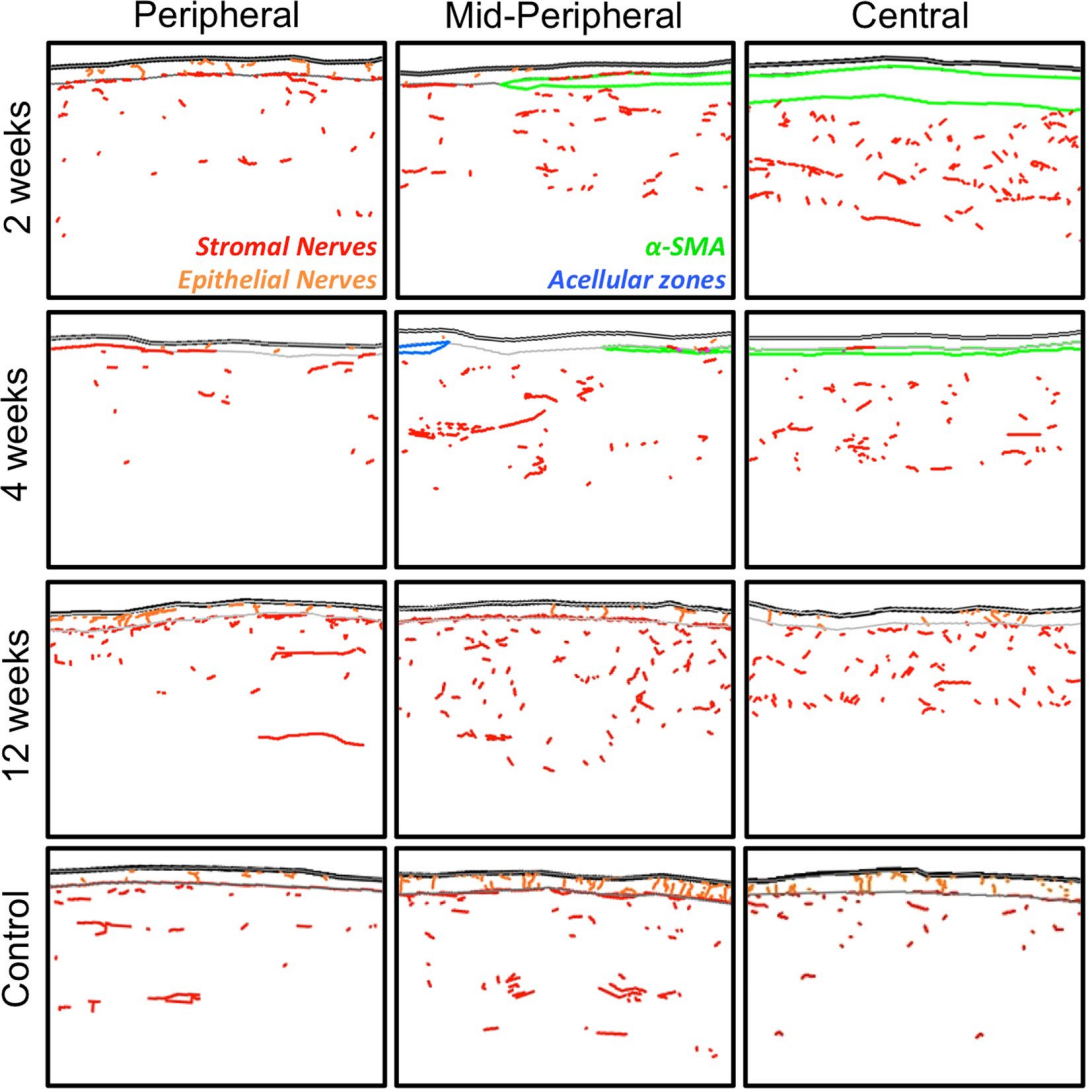
Sample tracings of immuno-stained corneal sections at different times post-PRK. The 3 columns show illustrative tracings from the peripheral, mid-peripheral and central corneas of 4 different cat eyes: first row, 2 weeks post-PRK; second row, 4 weeks post-PRK, third row, 12 weeks post-PRK and fourth row, unoperated control. Red and orange: Tuj-1 positive corneal nerves in stroma and epithelial layers, respectively. Green: regions positive for α-SMA. Blue: acellular zones. In all cases, the epithelium is shown at the top of each image.

The *in vivo* experiments described here were performed in cat eyes, so chosen because of structural and dimensional similarities between cat and human corneas, as well as because of the similarities in wound healing mechanisms and outcomes (biological and optical) between these two species^20–22,44^. As a baseline for assessing nerve regeneration after PRK, we first examined the [normal] distribution of nerves in different compartments of the intact, adult, cat cornea. The central feline cornea possesses a regular epithelium 7–9 layers thick and a stroma ~500 µm thick^20^ with no α-SMA positive zones (Fig. 1A). The anterior third to half of the stroma was populated sparsely by thick chords of Tuj-1 positive nerve fibers (red in Figs 1A,E, 2 and S1A), whereas as previously described^45^, the posterior third to half of the stroma was completely devoid of Tuj1-positive structures (Figs 1E and 2). Finer processes lined the basal layer of the epithelium, forming the sub-basal nerve plexus (pink in Figs 1A,E, 2 and S1A), running almost continuously from the periphery to the center of each corneal cross-section. Fine, Tuj-1 positive branches emerged from these sub-basal nerves, penetrating the epithelium almost perpendicularly (orange in Figs 1E, 2 and S1A), often spanning the entire epithelial thickness and ramifying into what looked like bare nerve endings within superficial epithelial layers. The density of epithelial nerves was lowest in the corneal periphery, increasing towards the central cornea.

**Figure 3:**
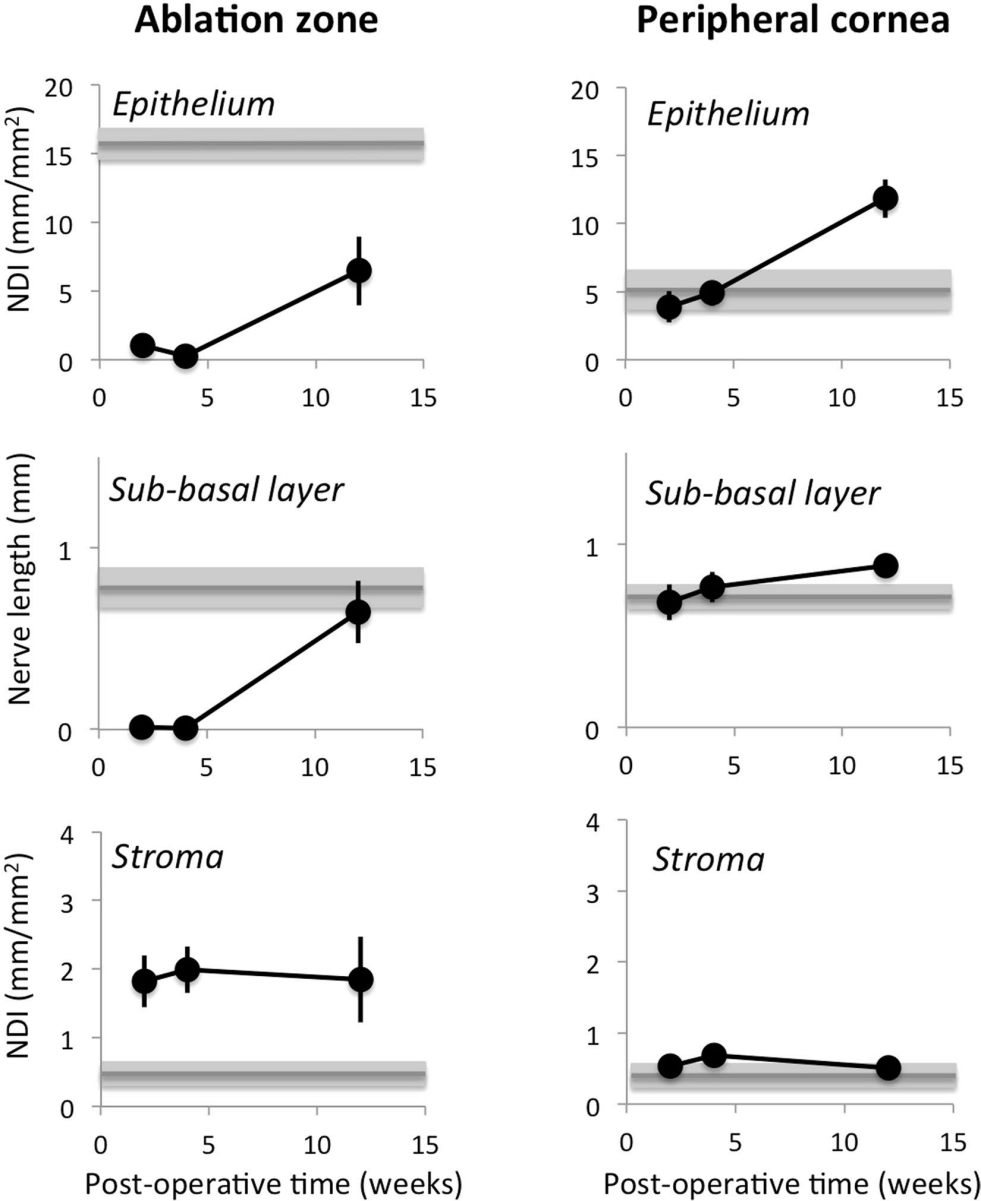
Quantitative analysis of nerve distributions in central and peripheral cat corneas post-PRK. Grey lines and shaded zones indicate mean ± standard error of the mean (SEM) of values obtained in normal, unoperated control corneas (n = 5). NDI: nerve density index. All data points are means ± SEM. See text for statistics.

Quantitative analyses (grey bars in Fig. 3) confirmed these observations, although we should note that the densities and nerve lengths provided here are of course confounded by the fact that analyses were done over portions of corneal cross-sections. As such, these numbers are not meant to be absolute, but serve only to provide a baseline for comparison between eyes at different post-operative times, and between corneal regions (central *versus* peripheral). In unoperated (i.e. normal) corneas’ center, the average Nerve Density Index (NDI) across the entire corneal thickness was 2.1 ± 0.6 (±SD) mm/mm^2^. For the stroma alone, NDI = 0.5 ± 0.07 mm/mm^2^, whereas the central epithelial NDI was much higher, at 15.7 ± 2.6 mm/mm^2^. In the periphery, total corneal NDI was 1.3 ± 0.2 mm/mm^2^; this was largely caused by the lower epithelial NDI of 5.1 ± 4.2 mm/mm^2^, while the stromal NDI remained at 0.4 ± 0.04 mm/mm^2^. The sub-basal nerve length contained within the standard analysis box also remained relatively similar between central and peripheral cornea, averaging 0.8 ± 0.2 mm in the center and 0.7 ± 0.1 mm in the periphery.

### Corneal injury dramatically and persistently alters corneal nerve distribution

PRK totally removed the epithelium and sub-basal layer over a central circular area about 8 mm in diameter. It also removed the anterior stroma (and all nerves it contained) over a depth of ~135 µm in a central 6 mm diameter circular zone. After PRK, the animals were allowed to heal and were euthanized at 2, 4 and 12 weeks post-PRK for histological analysis.

*Two weeks after PRK*, although a thin, Tuj-1-positive epithelium had regenerated across the entire ablation zone, there remained a complete absence of sub-basal and epithelial nerves in that zone (Figs 1B, 2 and 3; Fig. S1B; see Fig. S2 for labeling convention used throughout this manuscript). A strong, α-SMA-positive band (green in Figs 1B and 2) appeared in the stroma just under the healed epithelium. This band was also devoid of Tuj1-positive nerve fibers (Figs 1B, 2 and S1B). The extreme degree to which nerves seemed to “avoid” the α-SMA-positive zone was best illustrated at the edges of the ablation zone (middle column of panels in Fig. 2): sub-basal and epithelial nerves continued for a very short distance above the α-SMA zone, while stromal nerves were seen below it, but no nerves were seen inside it. In addition, the central stroma just below the α-SMA-positive zones contained >3 times the normal density of nerve fibers in that region (~1.8 *versus* 0.5 mm/mm^2^ in unoperated corneas, Figs 2, 3). Of note however, these excessively numerous fibers appeared to respect the distribution of stromal nerves seen in unoperated cat corneas, totally avoiding the posterior half of the stroma (Fig. S2). Finally, in contrast to changes in central corneas where the ablation zones were located, nerve distribution in all relevant compartments of the corneal periphery remained unchanged relative to controls, both qualitatively (Figs 1, 2) and quantitatively (Fig. 3).

*Four weeks after PRK*, the epithelium appeared healed and the thickness and extent of the αSMA-positive zones in the central cornea decreased markedly (Figs 1C, 2, S1C and S2A). Rarely, small, acellular zones appeared in the sub-epithelial stroma (blue in Figs 2 and S2A). In spite of a normal-appearing epithelial thickness however, the sub-basal and epithelial layers remained almost completely devoid of nerves in the central cornea, while maintaining essentially normal length (for sub-basal nerves) and NDI (for epithelial nerves) in the corneal periphery (Figs 2, 3). In contrast, just as was seen 2 weeks post-PRK, central stromal NDI was at least triple that in the normal, unoperated cat cornea, while peripheral stromal nerve density remained within the normal range (Figs 2, 3).

*By 12 weeks post-PRK*, corneas looked structurally normal in terms of epithelial and stromal thicknesses, with a total absence of α-SMA or acellular zones (Fig. 1D). Central epithelial NDI remained at about 50% of normal, averaging 6.5 ± 5.0 mm/mm^2^ (Figs 2, 3 and S1D). The large standard deviation suggests significant inter-ocular variability in regeneration behavior. In contrast, central sub-basal nerve length returned to within the normal range (0.7 ± 0.3 mm), with little variability between eyes (Fig. 3), leading us to conclude that inter-individual differences in central epithelial NDI may have resided primarily in the branching behavior of these sub-basal fibers into the epithelium. In the corneal periphery, however, epithelial NDI was more than double its normal value (Fig. 3), and this was accompanied by a slight increase in the length of underlying sub-basal nerves (0.9 ± 0.1 mm). All in all, a pattern of greater peripheral and variable but lower than normal central epithelial nerve density was seen 12 weeks after PRK. Finally, although α-SMA-positive zones were no longer present 12 weeks post-PRK, in 3 out of the 4 eyes analyzed, the central stroma continued to show a hyperdensity of nerves (Figs 2, 3). This was not due to a change in central stromal area analyzed (12 weeks post-PRK: 1.1 ± 0.1 mm^2^; control: 1.2 ± 0.3 mm^2^), but rather to a persistently high total length of Tuj-1 positive neural processes within this area (2.0 ± 1.3 mm) relative to the unoperated condition (0.5 ± 0.2 mm; two-tailed, independent sample t-test: *t*_5_ = *3.9*6, *p* = *0.011*). In contrast, stromal nerve density in the corneal periphery remained within the normal range, averaging 0.5 ± 0.4 mm/mm^2^.

In summary, even 12 weeks after PRK affecting only the central 6 mm of the ~16 mm diameter cat cornea, corneal nerve distribution failed to return to normal (Figs 1 and S1). Different abnormalities were seen in different compartments (epithelium, sub-basal layer, stroma) depending on corneal location (center or periphery). However, they invariably involved complete avoidance of regions and cells positive for α-SMA. This observation prompted us to develop an *in vitro* system which could be used to test the hypothesis that myofibroblasts inhibit neurite outgrowth in peripheral neurons, and to begin probing molecular signals mediating this effect.

### Robust neurite outgrowth in neuron/fibroblast but not neuron/myofibroblast co-cultures

**Figure 4:**
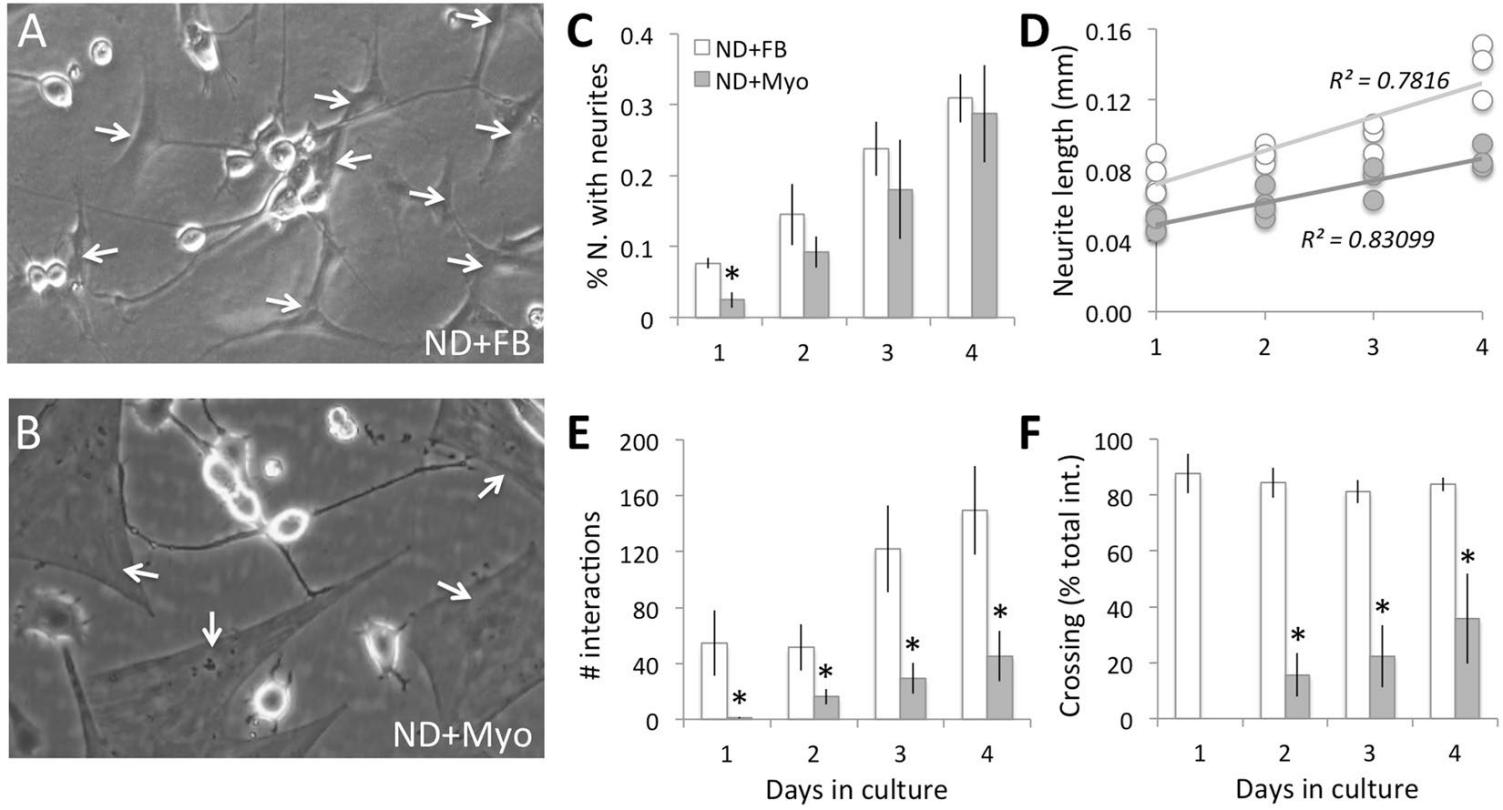
Differential effect on neurite growth of co-culturing ND7/23 cells with fibroblasts (FB) or myofibroblasts. (**A**) Phase contrast photograph of plated ND7/23 cells (highly refractile, round cell bodies) and FB (examples arrowed) after 4 days in culture. Note the long, thin neurites expressed by ND cells, which extend liberally across FB cell bodies and processes. (**B**) Phase contrast photograph of plated ND7/23 cells (highly refractile cell bodies) and Myo (examples arrowed) after 4 days in culture. Note the long, thin neurites expressed by ND cells, which exhibit distinct end-stopping when they contact Myos. (**C**) Plot of the percentage of plated ND7/23 cells with neurites >40 µm long at different times in either FB or Myo co-culture. While neuritogenesis appears slower in ND + Myo than ND + FB co-cultures at day 1, this difference decreases and disappears on subsequent days. (**D**) However, total neurite length/cell remained significantly greater in ND + FB co-cultures than in ND + Myo co-cultures at all time-points examined. (**E**) Plot of the total number of interactions (contacts) between ND cells and either FB or Myos. Note the persistently lower number of interactions between ND7/23 cells and Myos at all time-points, in spite of the fact that the same number of ND cells and Myos were plated initially. (**F**) Plot of the number of successful cell body crossings as a proportion of the total number of interactions between ND7/23 cells and either FB or Myo on different days in culture. When neurons contact a FB, they cross over its cell body and continue their extension >80% of the time. When ND cells contact a Myo, they tended to stop their advance; no crossings happened during Day 1 of culture, although they increased up to ~40% of the time by Day 4 in culture. All values are means ± SD from 3 separate experiments. *P < *0.05*. See Fig. S3A for sample Western blot made from 3 days old co-cultures in this experiment, confirming the presence of Tuj-1 positive cells in both co-culture types, the strong presence of α-SMA positive myofibroblasts in the ND + Myo co-cultures, and their total absence in ND + FB co-cultures.

To assess whether myofibroblasts did in fact possess the ability to inhibit neurite outgrowth in sensory neurons, we isolated cat corneal keratocytes and cultured them until they became fibroblasts, or using TGF-β1 stimulation, until they differentiated into myofibroblasts, as previously described^25^. We then seeded these fibroblasts or myofibroblasts with ND7/23 cells^26^. When neurite outgrowth was analyzed over the next 4 days, we found dramatic differences between these two co-culture systems. First, after 1 day, there was a significantly higher percentage of ND7/23 neurons with neurites >40 µm in ND/fibroblast (ND/FB) compared to ND/myofibroblast (ND/Myo) co-cultures (*t*_*6*_ = *3.02*, *p* = *0.023*, Fig. 4A). However, the percentage of neurons with neurites increased as a function of time in culture in both co-culture types, and after 4 days, the difference between co-cultures disappeared.

While neurite lengths also increased over time in both co-culture types (ND/FB: *r* = *0.88*, *t*_12_ = *6.51*, *p* < *0.0001*, CI_95_ upper and lower limits for rho = 0.663 and 0.962 respectively; ND/Myo: *r* = *0.913*, *t*_12_ = *7.74*, *p* < *0.0001*, CI_95_ upper and lower limits for rho = 0.741 and 0.972 respectively), those in ND/FB co-cultures were significantly greater than those in ND/Myo co-cultures at all time-points examined (Fig. 4D, paired t-test: *t*_13_ = *8.49*, *p* < *0.0001*).

In addition, when ND7/23 neurons were co-cultured with FBs, they contacted them liberally (Fig. 4A), with the number of interactions increasing linearly with days in culture (Fig. 4E). A different picture emerged in ND/Myo co-cultures: after 1 day in culture, ND cells did not extend long enough processes to actually contact Myos. From days 2–4 (Fig. 4E), contact occurred between the two cell types but the incidence remained far below that seen in ND/FB co-cultures. A two-way ANOVA showed a significant effect of co-culture type (FB *versus* Myo: *F(1,17)* = *58*.6*4*, *p* < *0.0001*), a significant effect of time in culture (*F(*2*,17)* = *13.65*, *p* = *0.000*8) and a significant interaction (*F(2,17)* = *4.56*, *p* = *0.034*).

Finally, the nature of the interactions between ND cells and FB or Myos also differed. When neurons contacted FBs, they crossed over the cell bodies and continued their extension >80% of the time (Fig. 4A). When ND cells contacted Myos, they tended to stop their advance (Fig. 4B) and crossed the Myo cell bodies at a much lesser rate than was seen for FBs (Fig. 4F). No crossings were seen during the first day of ND/Myo co-culture because there were no cell contacts at that time (see Fig. 4E,F). From days 2–4, the incidence of ND neurites growing across Myo cell bodies increased from about 16% to 36% of all interactions observed (Fig. 4F), consistently lower than the incidence of crossings in ND/FB co-cultures at the same time points. A two-way ANOVA revealed a significant effect of co-culture type (FB versus Myo: *F(1,17)* = *179.49*, *p* < *0.0001*), but no significant effect of time in culture (*F(2,17)* = *2.1*8, *p* = *0.156*) or interaction (*F(2,17)* = *1.9*, *p* = *0.192*).

### Myofibroblast inhibition of neurite outgrowth in culture is TGF-β receptor dependent

**Figure 5:**
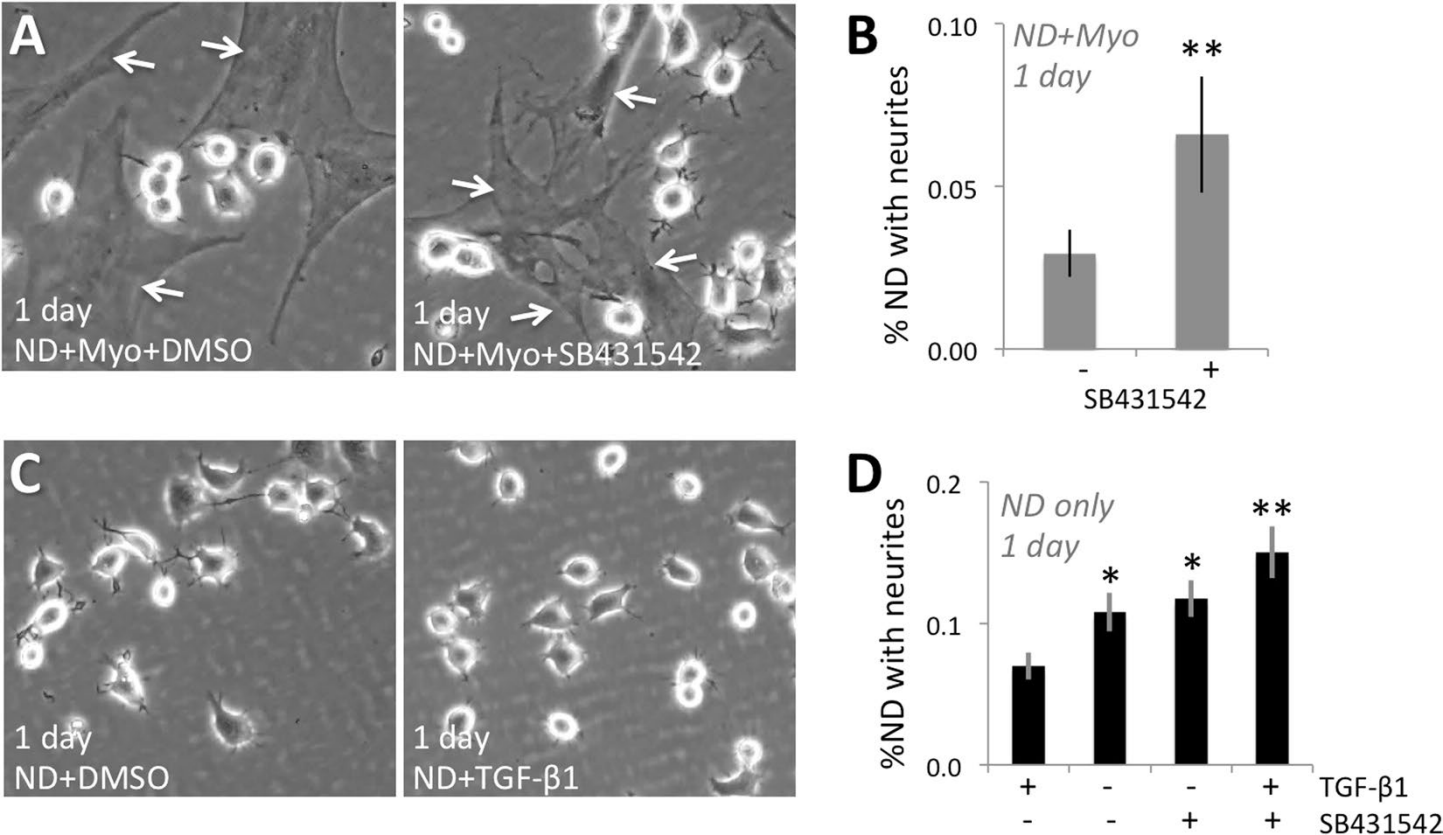
Critical role of TGF-β1 and its receptor in mediating the inhibitory effect of myofibroblasts (Myos) on neurite outgrowth in 1 day old cultures. (**A**) Phase contrast photograph of plated ND7/23 cells (highly refractile, round cell bodies) and co-cultured Myos (examples arrowed) in the presence of DMSO or the TGF-β receptor inhibitor SB431542. Note the rare neurites expressed by ND7/23 cells after 1 day in co-culture with Myos and DMSO, in contrast with the numerous neurites expressed by ND cells, in the same co-culture, when SB431542 is present. (**B**) Plot of the percentage of plated ND7/23 cells with neurites >40 µm long in ND + Myo co-cultures in the presence or absence of SB431542. See Fig. S3B for sample Western blot made from co-cultures in this experiment, confirming the presence of similar amounts of Tuj-1 and of α-SMA positive myofibroblasts irrespective of treatment with SB431542. (**C**) Phase contrast photograph of ND7/23 cells (highly refractile, round cell bodies) in pure cultures in the presence of DMSO or TGF-β1. Note the multiple neurites expressed by ND7/23 cells after 1 day in culture with DMSO, and the marked decrease in neurites when ND cells are treated with TGF-β1. (**D**) Plot of the percentage of plated ND7/23 cells with neurites >40 µm long when treated with or without TGF-β1. The dependence of this effect on activation of the TGF-β receptor is demonstrated by the ability of the TGF-β receptor inhibitor SB431542 to eliminate the anti-neuritogenic impact of TGF-β1. See Fig. S3C for sample Western blot made from mono-cultures in this experiment, confirming the presence of similar amounts of Tuj-1 irrespective of treatment with DMSO, TGF-β1, SB431542 or TGF-β1 + SB431542. All values in graphs are means ± SD from 3 separate experiments. * *P* < *0.05*, **P < *0.01*.

After 1 day (Fig. 5A,B), the percentage of ND cells with neurites >40 µm long was twice as large when they were co-cultured with Myos + SB431542 (TGF-β receptor inhibitor) *versus* Myos + DMSO (vehicle); two-tailed, independent sample t-test: *t*_*6*_ = *3.8*, *p* = *0.009*. In fact, addition of the TGF-β receptor inhibitor restored the proportion of neurons with neurites in ND/Myo co-cultures back to levels seen in 1-day old ND/FB co-cultures (Fig. 5A,B). This suggested that the inhibitory actions of Myos on neurite outgrowth may be mediated by their secretion of TGF-β1. TGF-β1 is the strongest-known pro-fibrotic molecule, whose secretion is dramatically upregulated in corneal wounds^46,47^. Of note, TGF-β1, which is used to differentiate FBs into Myos *in vitro*, was washed out of the cultures before adding ND7/23 cells. Thus, in our co-cultures, Myos were the only possible source of this pro-fibrotic molecule.

### TGF-β1 mimics inhibitory effect of myofibroblasts on neurite outgrowth

In a further test of our hypothesis, ND cells were cultured alone (i.e. without FBs or Myos) for 1 day in the presence of DMSO (control), DMSO + TGF-β1, DMSO + TGF-β1 + SB431542 or DMSO + SB431542 (Fig. 5C,D). A one-way ANOVA performed with respect to the proportion of ND cells expressing neurites >40 µm in length revealed a significant effect of treatment *(F (3,23)* = *6.03*, *p* = *0.0043*), supporting the notion that TGF-β1 decreased neurite outgrowth relative to the control conditions. Critically, a post-hoc Tukey HSD test showed that TGF-β1 + SB431542 significantly increased the proportion of cells with neurites relative to the TGF-β1 condition (p < 0.01), confirming that TGF-β1’s effects were mediated by the TGF-β receptor.

### TGF-β1 increases p-CRMP2 expression in pure ND7/23 cultures

**Figure 6:**
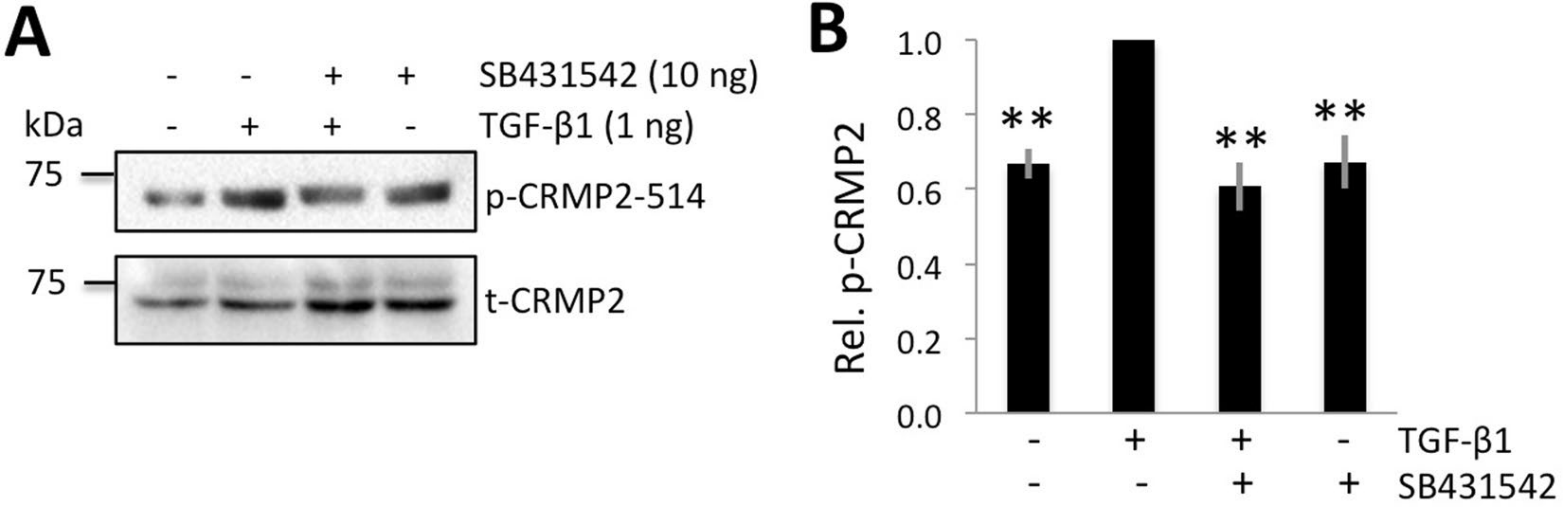
TGF-β1 increases relative p-CRMP2 expression in pure ND7/23 cultures. (**A**) Representative Western blots showing protein levels for phosphorylated (p-) CRMP2 and total (t-) CRMP2 in cultured ND7/23 cells with or without TGF-β1 stimulation. The samples were run on separate blots. Basal levels of both p- and t-CRMP2 were distinctly above zero. After 1 hour, TGF-β1 increased the expression of p-CRMP2, but not that of t-CRMP2. The TGF-β receptor inhibitor SB431542 blocked the up-regulation of p-CRMP2 observed following TGF-β1 stimulation. (**B**) Plot of relative expression of p-CRMP2/t-CRMP2 normalized to densitometric values obtained in cells treated with TGF-β1. Data shown are averaged over three experiments. Total CRMP2 levels were were used as loading controls. Data are expressed as means ± SD. **P < *0.01* versus the TGF-β1 only condition. Full, unedited gels analyzed are shown in Supplementary Figure 5 (Fig. S5).

Having established that the inhibitory effect of corneal myofibroblasts on neuritogenesis is likely mediated by TGF-β1, our final question in this series of experiments was to begin probing the signaling mechanisms in sensory neurons, which mediate this effect. We first used immunohistochemistry to confirm that feline corneal nerves (as well as stromal keratocytes) normally express CRMP2 (Fig. S2). Western blots (example in Fig. 6A,B) were then used to show that in ND7/23 monocultures, TGF-β1 increased levels of p-CRMP2 while leaving total levels of CRMP2 unaffected. On average, TGF-β1 caused the p-CRMP2 (514)/t-CRMP2 ratio to increase 1.5 ± 0.1 fold over baseline (two-tailed paired sample t-test between baseline and TGF-β1: *t*_*2*_ = *24.42*, *p* = *0.0017*, CI_95_ for difference of means = ± 0.072). This effect appeared to be entirely mediated by the TGF-β receptor since addition of SB431542 abolished TGF-β1’s ability to increase p-CRMP2 expression (Fig. 6A,B). In fact, the p-CRMP2/t-CRMP2 levels of ND cells treated with TGF-β1 + SB431542 or SB431542 alone were not significantly different from baseline (one-way ANOVA, *F (2,8)* = *1.06*, *p* = *0.403*)

### Blocking myofibroblast differentiation enhances corneal nerve regeneration *in vivo*

**Figure 7:**
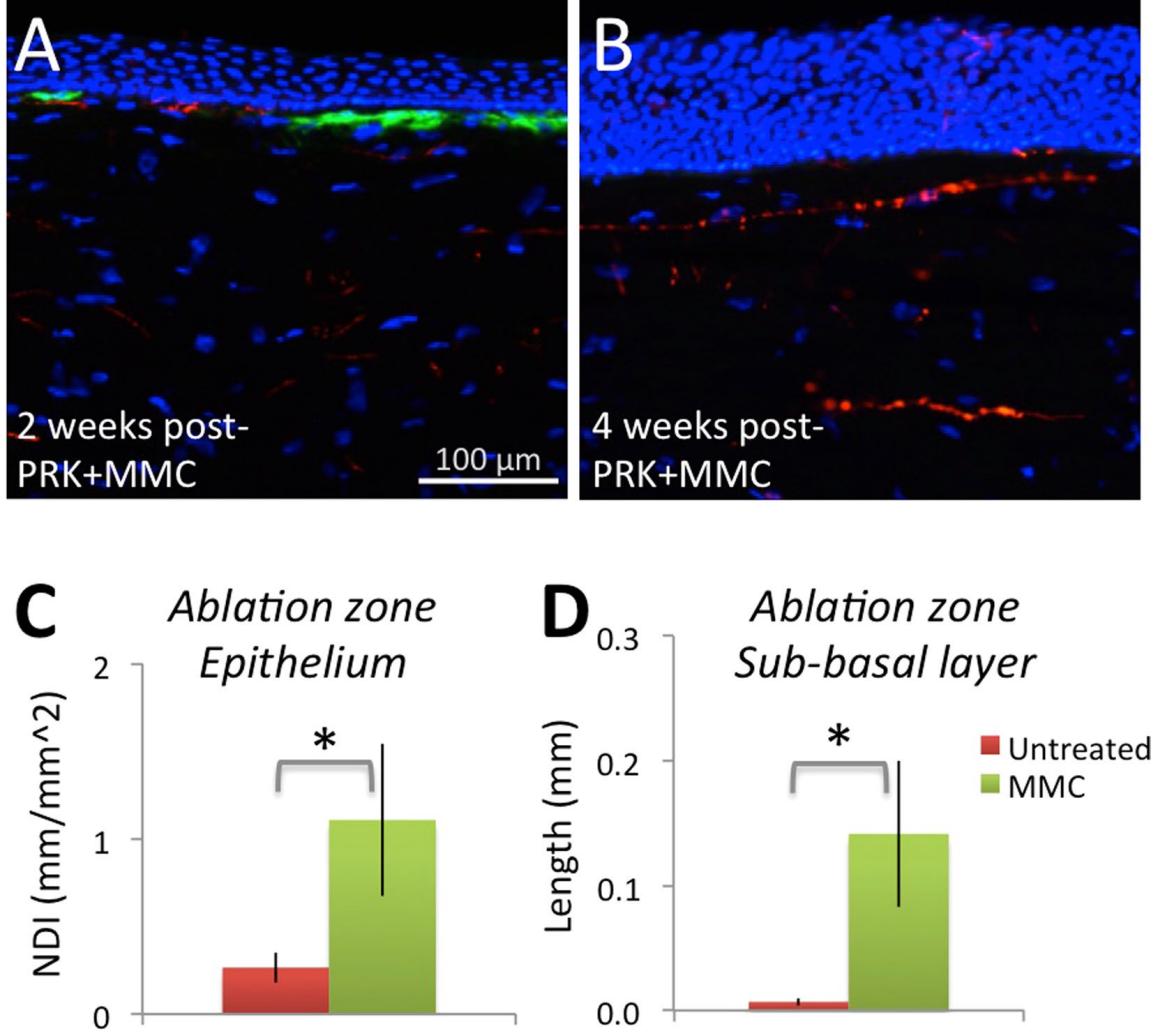
Immunohistochemical staining and analysis of feline corneas treated with PRK + MMC. (**A**) Photograph of the central cornea of a cat 2 weeks after PRK + MMC reacted for Tuj-1 (red fluorescence), α-SMA (green fluorescence) and counter-stained with DAPI (blue fluorescence). Note the thin zone of positive α-SMA staining, which is adjacent to a zone devoid of α-SMA staining and which contains nerves. Note also the thin, densely distributed stromal nerves, and the fully-regenerated epithelium. (**B**) Photograph of the central cornea of a cat 4 weeks after PRK + MMC, stained identically as in A. Note the total absence of an α-SMA positive zone, which contrasts with that seen in Fig. 1. The epithelium is markedly hyperplastic and contains some sparse nerves. (**C**) Plot of mean ± SEM epithelial NDI (nerve density index) at 4 weeks post-PRK in cat eyes treated with MMC or untreated. There are significantly more corneal nerves in the epithelium following MMC treatment than in untreated corneas (*p < *0.05*). (**D**) Plot of mean ± SEM sub-basal nerve length at 4 weeks post-PRK in cat eyes treated with MMC or untreated. There are almost no sub-basal nerves in the ablation zone of cats with PRK only. There are significantly more corneal nerves in the sub-basal layer of the ablation zone 4 weeks after PRK + MMC treatment (*p < *0.05*).

As a final test of our hypothesis that myofibroblasts exert a biologically significant inhibitory effect on regenerating corneal nerves, we used an intra-operative application of Mitomycin C (MMC) to decrease myofibroblast differentation after PRK^40–43^ in 8 eyes from 4 cats. Two and four weeks after PRK + MMC, we euthanized the cats, excised the eyes and processed corneal sections histologically as described earlier. Histologically, there were dramatic differences between MMC-treated (Fig. 7A,B) and non-treated eyes (Fig. 1A–D) with markedly less α-SMA positive staining at 2 weeks and none at 4 weeks post-PRK. In fact, at 2 weeks post-PRK, the α-SMA positive layer was thin and interrupted by α-SMA negative zones populated by Tuj-1 positive nerves (Fig. 7A). By 4 weeks, it was possible to see the impact of dampening myofibroblast differentiation on nerves: though by no means back to normal, both intra-epithelial nerve densities (Fig. 7C) and the length of sub-basal nerves (Fig. 7D) in the ablation zone were several fold higher in MMC-treated compared to untreated eyes post-PRK (epithelial NDI *t*_*8*_ = *2.35*, *p* = *0.0467*; sub-basal length *t*_*8*_ = *2.88*, *p* = *0.0205*).

## Discussion

The present study is the first to systematically describe an active inhibitory effect of corneal myofibroblasts, but not fibroblasts, on regenerating corneal nerves after injury. First, we showed that *in vivo*, in a large mammalian cornea, regenerating stromal nerves over-populate the sub-ablation stroma, but completely avoid thick regions of stroma expressing α-SMA, a marker of differentiated myofibroblasts^46^. This avoidance behavior was observed both at 2 and 4 weeks post-PRK, and could be inferred to exist whenever α-SMA is expressed *in vivo*. A consequence of this avoidance was that above α-SMA positive zones, there were no sub-basal nerves and the regenerated epithelium remained completely devoid of intra-epithelial nerves. Thus, it seemed that α-SMA positive zones *prevented* stromal nerves in the central cornea from repopulating the sub-basal and epithelial layers above them. While peripheral nerves appeared normally distributed in all compartments at 2 and 4 weeks post-PRK, they did not repopulate the sub-basal layer and epithelium above the α-SMA positive zones. Though speculative, one interpretation of this finding is that one month post-PRK, a relatively large area of central cornea several mm in diameter remained either devoid of sensation or with dulled/abnormal sensation^48–50^.

Even once α-SMA-positive zones disappeared, central epithelial nerves appeared to regenerate slowly, remaining at 50% of normal by 12 weeks post-PRK, again in spite of a fully regenerated, structurally normal epithelium since 4 weeks. The cornea peripheral to the ablation zone remained unremarkable until 12 weeks post-PRK, when peripheral epithelial nerve density grew to about double normal values, while sub-basal and stromal nerve densities remained within the normal range. The sensory implications of these abnormal epithelial nerve densities across the healed cornea remain to be determined; one could speculate that increased nerve densities in the peripheral epithelium may engender unpleasant, painful and/or increased sensation within this region^2,48,50–52^.

Our *in vivo* observations raised many questions – first, what was it about α-SMA-positive zones that prevented regenerating stromal nerves from crossing into them? What cells within the α-SMA-positive zones were responsible for this phenomenon? Was the effect due to contact inhibition, a releasable factor or both? And finally, what molecules and signaling pathways mediated these effects on nerves?

Our first hypothesis was that myofibroblasts themselves were responsible for the apparent growth-stopping effects of α-SMA positive zones on regenerating nerves. However, regions of the corneal stroma positive for α-SMA after PRK do not just contain myofibroblasts, but can include neutrophils, macrophages/monocytes, T cells and bone-marrow derived cells^53,54^. In mouse, bone-marrow derived cells are also capable of differentiating into myofibroblasts^55,56^. To test whether corneal keratocytes themselves, when stimulated to differentiate into fibroblasts and myofibroblasts, are capable of exerting an inhibitory action on growing nerves, we needed to isolate and test them independent of other corneal cell types. As such, we isolated, cultured and differentiated primary feline corneal keratocytes into either fibroblasts or myofibroblasts, as previously described^22,25,57,58^. We then seeded these fibroblast or myofibroblast cultures with ND7/23 cells. Co-cultures were photographed and analyzed over 4 consecutive days, allowing us to observe and quantify dramatically different neuronal behavior when ND7/23 cells were co-cultured with myofibroblasts as opposed to fibroblasts. Our findings with respect to ND/FB co-cultures were consistent with recently published results showing that corneal fibroblasts secrete a battery of factors, which promote neurite outgrowth in dorsal root ganglion cells^19^. In contrast, myofibroblasts appeared to actively inhibit neurite outgrowth when co-cultured with ND7/23 cells. The differences between fibroblast and myofibroblast/ND co-cultures could be summarized as follows: the onset of neurite outgrowth appeared delayed and the rate of elongation was slowed in ND/Myo compared to ND/FB co-cultures; once neurites became long enough to contact myofibroblasts, they exhibited contact inhibition, whereas they grew readily across fibroblast cell bodies and processes.

The fact that co-culturing ND7/23 cells with myofibroblasts delayed the onset of neurite outgrowth and slowed neurite elongation *before* cell-cell contact occurred suggested that the effects of myofibroblasts on neuritogenesis were likely mediated by one or more releasable factors. Both fibroblasts and myofibroblasts synthesize and release multiple cytokines^59–62^ but myofibroblasts are also known to secrete TGF-β1^37,63,64^. Given the abundance of this molecule in the wound environment^65^, we asked: (1) could TGF-β1 actually be the factor released by myofibroblasts, which decreases neuritogenesis in our co-culture system, and (2) how do sensory neurons process the TGF-β1 signal? Given our finding that the TGF-β receptor blocker SB431542 restored the percentage of cells with neurites >40 µm (our criterion) in ND/Myo co-cultures to levels observed in ND/FB co-cultures, we concluded that the inhibitory effect of myofibroblasts on neurite outgrowth was mediated by activation of the TGF-β receptor. We then generated pure cultures of ND7/23 cells and exposed them to TGF-β1. This treatment mimicked the effect of co-culturing ND7/23 cells with myofibroblasts, almost halving the percentage of neurons with neurites >40 µm relative to the control condition, and also via the TGF-β receptor. Thus, our results strongly suggest that myofibroblasts in ND/Myo co-cultures secrete TGF-β1, which inhibits neurite outgrowth in ND7/23 cells. Please note that *in vivo*, the damaged epithelium is thought to be a major source of TGF-β1 post-PRK^66^, and it may also contribute to the inhibition of nerve regeneration in the early post-surgical period. However, our focus on myofibroblasts in the present study was motivated by the observation that even 2–4 weeks after PRK, when the epithelial basement membrane had presumably been re-established^66^, blocking access to epithelial-derived TGF-β1, nerves continued to be absent from the epithelium, the sub-basal layer and a fairly large region of the anterior stroma populated by myofibroblasts.

We next asked whether TGF-β1’s effect on neuritogenesis in ND7/23 cells was mediated by CRMP2 signaling. As mentioned earlier, CRMP2 is a likely candidate molecule regulating axonal guidance and neuronal polarity^37^. Importantly, we now show that CRMP2 is expressed in corneal nerves *in vivo* (Fig. S4). If myofibroblasts inhibit neurite outgrowth in co-cultured ND7/23 cells by secreting TGF-β1, which inactivates CRMP2, then *monocultures* of ND7/23 cells exposed to TGF-β1 should have increased expression of the phosphorylated form of CRMP2 (p-CRMP2), and this should be blocked by addition of the TGF-β receptor inhibitor SB431542. Both phenomena were observed in the present set of experiments, suggesting that changes in CRMP2 signaling in ND7/23 cells do indeed correlate with changes in neurite outgrowth, and that they are controlled by TGF-β1 binding to, and activating its receptor. However, while neurite initiation is slowed by TGF-β1 and by co-culturing with myofibroblasts, it is not completely abolished. Future studies will investigate whether other releasable factors work together with TGF-β1 and additionally, what cell signaling mechanisms underlie the contact inhibition observed in later stage ND/Myo co-cultures.

Finally, to directly test our premise that scar-forming myofibroblasts inhibit nerve regeneration, we returned to our cat model of PRK-induced corneal injury and asked whether dampening the fibrotic aspects of wound healing with an intra-operative application of MMC improved the rate of corneal nerve regeneration. Two weeks after PRK, we verified that MMC did in fact significantly decrease the amount of α-SMA positive staining under the fully-regenerated epithelium. The band of α-SMA positive cells in the ablation zone was very thin and interrupted, with those interruptions populated by nerves. By 4 weeks after PRK, MMC-treated corneas had no α-SMA positive zones; some sub-basal nerves appeared to have returned, albeit not quite to normal levels, and intra-epithelial nerves were also partially restored. This partial restoration of nerves in the ablated cornea across all compartments that normally contain them is extremely encouraging, and shows that our premise about the negative influence of myofibroblasts is likely correct. This is in spite of the fact that MMC is not a perfect anti-fibrotic solution for corneal wounds. Indeed, it works by indiscriminately killing cells in the exposed stromal bed, creating large acellular zones (blue zones in Fig. S2B). This is important because some of the cells killed include those beneficial to regrowing nerves, such as fibroblasts^19^. Nonetheless, our MMC data offer the promise of even better outcomes if future studies can use more specialized pharmacologics, such as peroxisome proliferator-activated receptor gamma ligands^22,25,57^ to better target myofibroblast differentiation, with a less negative impact on other cells or aspects of corneal wound healing.

In summary, our results represent a conceptual advance in understanding the complex interactions that need to be controlled to achieve successful healing of corneal, and likely other types of wounds. In addition, they have significant practical and clinical implications. First, they suggest that blocking myofibroblast differentiation should be considered a critical component of therapies that aim to restore normal corneal innervation in conditions such as neurotrophic keratitis or laser refractive surgery, where nerves are damaged and corneal wound healing leads to the appearance of this cell type^67^. Second, identification of signaling mechanisms that mediate myofibroblast inhibition of neurite outgrowth could provide alternative therapeutic targets to allow nerves to regenerate *even in the presence of differentiated myofibroblasts*. This could be especially beneficial when patients present long enough after damage has occurred, and fibrosis or scarring are already present in the cornea. It would also be beneficial if in fact, it became important to preserve the positive contribution of myofibroblasts for corneal wound healing (i.e. protecting the cornea from perforation in the context of infected injuries^68^).

## Methods

### *In vivo* experiments

All animal procedures were conducted according to the guidelines of the ARVO Statement for the Use of Animals in Ophthalmic and Vision Research, and the NIH Guide for the Care and Use of Laboratory Animals. The protocol was approved by the University of Rochester Committee on Animal Research (UCAR, Assurance Number: A-3292-01). All cats were obtained from a research breeding colony managed by Liberty Research Inc. (Waverley, NY, USA). Relevant to the health of corneal nerves specifically, all cats and cat tissues obtained for this study came from animals either vaccinated with a killed vaccine that covers feline panleukopenia, rhinotracheitis (herpes virus) and calici (Fel-O-Vax PCT + Calicivax) or confirmed sero-negative for Herpes Simplex Virus serotype I (HSV-1) and maintained in isolation. Photorefractive keratectomies (PRK) were performed first on 16 eyes from young (1–2 yrs old) adult, domestic, short hair cats (*felis cattus*), which were sacrificed for histology at 2 (n = 6), 4 (n = 6), and 12 weeks (n = 4) post-PRK. Five additional eyes were obtained to serve as unoperated, untreated, normative controls. Finally, 8 eyes from 4 cats underwent PRK followed by intra-operative application of MMC.

#### Laser ablation

PRK was performed with a commercial excimer laser (Technolas 217, Bausch & Lomb Inc.) on the center of each cat cornea, following manual debridement of the epithelium over a circular area about 8–9 mm in diameter. A 10 diopters (D) myopic ablation was performed over a 6 mm optical zone (OZ), generating a central, stromal ablation depth of about 135 µm (Zyoptix 4.14; Bausch & Lomb Inc.). Surgeries were performed by the same refractive surgeon (HH) under topical (Proparacaine 0.5%, Falcon) and surgical anesthesia (Ketamine, 5 mg/kg, Dexmedetomidine Hydrochloride 0.04 mg/kg). Immediately after PRK, the first 16 cat eyes were immediately rinsed with balanced salt solution (BSS, Alcon Inc.) followed by a drop of antibiotic solution (Neomycin, Polymyxin B Sulphate, Gramicidin Ophthalmic Solution USP, Bausch & Lomb Inc.) twice daily for 2 weeks post-PRK. In the case of the last 8 cat eyes, 0.02% MMC (Mobius Therapeutics, LLC, St Louis, MO) was applied intra-operatively and held in place on the ablated stromal bed using a saturated, sterile, gelatin sponge (SurgifoamTM; Ethicon) for 1 minute immediately post-PRK, followed by copious irrigation with BSS. Antibiotic treatment was then administered as described above.

#### Histology and immunohistochemistry

Cats were euthanized and their corneas were excised, and immersion fixed in 1% paraformaldehyde in 0.1 M phosphate buffered saline (PBS), pH 7.4 for 10 minutes. They were then cryoprotected in 30% sucrose in 0.1 M PBS at 4 °C for 2 days. The tissue was then embedded in Tissue Tek O.C.T. Compound (Sakura Finetek) and frozen. Serial 20 µm, transverse sections were cut using a cryostat (2800 Frigocut E, Leica), collected three at a time on gelatin-coated glass slides, and stored at −20 °C until stained. For corneas that underwent PRK, one in every ten slides was first stained with H&E using standard protocols to identify the ablation zone, which was roughly in the center of each cornea. Slides containing sections close to the ablation center of PRK corneas or the geometric center of unoperated corneas were then selected for immunohistochemistry, targeting alpha-smooth muscle actin (α-SMA, staining myofibroblasts) and beta-tubulin (staining corneal nerves). Sections were first co-incubated with a rabbit polyclonal anti-α-SMA αantibody (ab5694 Abcam, Cambridge, MA; 1:400) and a mouse monoclonal anti-Tuj1 antibody (MMS-435P, Covance, Princeton, NJ; 1:1000) overnight at 4 °C. Some sections from each treatment group were also incubated overnight with only PBS containing 0.1% Triton X-100 as a negative control. After rinsing, secondary antibodies were applied to the sections: Alexa Fluor 488 conjugated to goat anti-rabbit IgG (A11008, Invitrogen, Grand Island NY; 1:400) and Alexa Fluor 555 conjugated to goat anti-mouse IgG (A21422, Invitrogen, Grand Island, NY; 1:400). They were incubated at room temperature for 2 hours, rinsed and cover-slipped with Vectashield Mounting Medium containing DAPI (Vector Laboratories, Burlingame, California). This generated a set of triple-labeled sections: Blue fluorescence (DAPI)-labeled cell nuclei, Green fluorescence (Alexa 488)-labeled α-SMA, and Red fluorescence (Alexa 555)-labeledTuj1 indicating nerves.

#### Analysis of corneal nerve distributions

The distribution of corneal nerves was assessed in 4 different corneal sub-regions: the epithelium, sub-basal layer, stroma and regions of α-SMA staining (containing myofibroblasts). Four to six immunostained sections from at least 4 different eyes were selected for analysis at each time-point. Stained sections were first imaged using an Olympus BX53 microscope equipped with a motorized stage and interfaced through a Q Imaging camera (Quantitative Imaging Corporation, Surrey, BC, Canada), with a computer workstation running the Neurolucida software (MicroBrightField Biosciences, Williston, VT). Pictures were first collected using a 10x objective and the appropriate fluorescent cubes (DAPI, FITC, and TxRED - Semrock, Rochester,NY). The captured images were traced manually in Neurolucida, outlining the entire corneal section with a closed contour. The process was repeated to generate a contour around the epithelium, and any α-SMA-positive zones (if present). Each Tuj1-positive nerve fiber was then traced, and color-coded to indicate segments that passed through the epithelium, the sub-basal layer, and zones of the stroma that were either normal or α-SMA positive (see Fig. 1 and S2 for labeling schemes).

Quantitative analysis was performed over a box of constant size (2390 µm × 1350 µm) placed in the approximate center of the ablation zone and in the mid-periphery (half-way between the outer edge of the ablation zone and the edge of the cornea) of each drawn section. In all cases, the drawn section was first rotated in Neurolucida until it was aligned to the auto-move box and the “magnification” was reduced to 4x. The box was drawn over the region of interest (ablation center or periphery) and the corneal sub-regions contained with the box (epithelium, stroma, regions of α-SMA staining) were outlined manually so that their areas could be measured. The length of nerves in each of the sub-regions inside a given box were automatically computed and exported using Neurolucida Explorer (MicroBrightField Biosciences, Williston VT) into an Excel spreadsheet, where we recorded the total nerve length in the sub-basal layer, and computed a nerve density index (NDI) for the epithelium and stroma. The NDI was calculated by dividing total nerve length within the epithelium or stroma (L_e_ or L_s_) by the area of the epithelium or stroma within the box (A_e_ or A_s_):

$${{\rm{NDI}}}_{{\rm{e}}{\rm{or}}{\rm{s}}}={\rm{total}}\,{{\rm{L}}}_{{\rm{e}}{\rm{or}}{\rm{s}}}/{{\rm{A}}}_{{\rm{e}}{\rm{or}}{\rm{s}}}$$

All values were then averaged across at least 4–6 sections/eye, for all eyes in each group and time-point.

### *In vitro* experiments

#### Co-cultures of cat corneal fibroblasts and ND7/23 cells

Primary feline corneal fibroblasts were generated as previously described^25^. Corneas were dissected post-mortem from young, adult, domestic short-hair cats (*felis cattus*). The epithelium and endothelium were removed and the stroma underwent double enzyme digestion. Isolated cells were grown in fibroblast growth factor (FGF) medium (PromoCell GmbH, Germany), refreshed every second day until they reached confluence. After passage 2, the medium was changed to DMEM/F12 (Cellgro^TM^, Manassas, VA) with 5% of mitogen-poor horse serum (HS – catalog # P5552; Sigma Aldrich, St. Louis, MO) to keep cells in a quiescent state^69–72^. Cells were passaged 3–7 times to generate sufficient numbers for experiments, but because they were cultured in 5% HS and cell density was maintained within a consistent range, the resulting cultures were relatively free of α-SMA (see western blots in Fig. S3A), and thus myofibroblasts. From these pure corneal fibroblast cultures, we generated either fibroblast/neuron or myofibroblast/neuron co-cultures as follows: a neuronal hybridoma cell line ND7/23 (Sigma Aldrich, St. Louis, MO) was plated in DMEM/F12 (Cellgro^TM^, Manassas, VA) with 10% of fetal bovine serum (FBS; Sigma) and multiplied until passage 25.

To generate fibroblast-ND7/23 co-cultures, passage 3–5 fibroblasts were seeded at a density of 1.5 × 10^5^/6-cm dish in DMEM/F12 + 5% HS + 0.5% FBS. The medium was then changed to DMEM/F12 + 0.5% FBS for 1 day. Separately, ND7/23 cells were also placed in DMEM/F12 + 0.5% FBS for 1 day before adding them to the fibroblasts. To generate myofibroblast-ND7/23 co-cultures, passage 6–7 corneal fibroblasts were seeded at a density of 1.5 × 10^5^/6-cm dish in DMEM/F12 + 5% HS + 0.5% FBS. This was changed to DMEM/F12 + 0.5% FBS for 1 day to promote quiescence before adding 1 ng/ml recombinant human TGF-β1 (R&D Systems Inc., Minneapolis, MN) and incubating for another 3 days. This treatment was repeated until days 10–12 to ensure that ~90% cells became myofibroblasts^73,74^, as verified by positive α-SMA staining. At this stage, 8 × 10^4^ ND7/23 cells were added to each dish and co-cultures were fed 50 ng/ml of Nerve Growth Factor (NGF, R&D Systems Inc.) on days 0 and 2, kept until day 5. All cell culture experiments were performed at least in triplicate.

#### Quantitative analysis of neurite outgrowth in culture

Phase-contrast images of each co-culture dish were captured using an inverted Olympus IX73 microscope (Center Valley, PA) under 10X magnification. A 19-hole imaging template was used to standardize sampling of each dish and photography was performed at days 1, 2, 3 and 4 in co-culture. Imaging software (Olympus CellSens Standard ver. 1.12, Olympus) was used to manually count the number of cells with neurites >40 µm in length (two fold longer than the average diameter of an ND7/23 cell soma), as well as to trace and measure total neurite length/cell. Neurite length was defined as the length from the cell soma to the tip of the neurite. We also examined interactions between ND7/23 cells and fibroblasts or myofibroblasts. First, the total number of neuron-to-fibroblast/myofibroblast contacts was counted in each image. Then, each contact was classified according to the neurite’s behavior: a neurite was defined as “crossing” a fibroblast/myofibroblast if it traversed at least a fifth of the contacted cell’s surface; it was defined as “ending” when its tip stopped or turned aside without crossing the cell at the point of contact. Experiments were repeated three times using co-cultures prepared on separate days.

#### Effect of TGFβ receptor inhibitor on neurite outgrowth in ND/Myo co-cultures

Myofibroblasts were pretreated with either the TGFβ receptor inhibitor SB431542 (10 ng/ml) in DMSO or DMSO alone (control) for 30 min before adding differentiated ND7/23 cells (separately cultured in DMEM/F12 + 0.5% FBS for 1 day) at a density of 8 × 10^4^ cells/6-cm dish together with 50 ng/ml NGF. After 24 hours, 19 fields per co-culture dish were imaged using an inverted, phase-contrast microscope (see above) and the percentage of ND7/23 cells with at least one neurite >40 µm long was computed as a function of the total number of cells on each plate.

#### Effect of TGF-β1 on neurite outgrowth in ND7/23 mono-cultures

ND7/23 cells were first grown in DMEM/F12 + 0.5% FBS for 1 day before seeding at a density of 8 × 10^4^ cells/6-cm dish together with either 50 ng/ml NGF or 10 ng/ml TGF-β1 + 50 ng/ml NGF. After 24 hours, 19 fields per dish were imaged using an inverted, phase-contrast microscope, as described earlier, and the percentage of ND7/23 cells with at least one neurite >40 µm long was computed as a function of the total number of cells on each plate.

#### Effect of TGF-β1 on p-CRMP2 expression in ND/23 cells

ND7/23 cells were cultured in DMEM/F12 containing 0.5% FBS overnight. The next day, the neurons were seeded at a density of 3 × 10^5^ cells/6-well plate containing DMEM/F12 + 0.5% FBS + 3.7 µM NGF. After attachment, cells were pretreated with 26 nM SB431542 for 30 min before adding 1 ng/ml TGF-β1 for 1 hour. Cells were then harvested and a polyclonal rabbit anti-p-CRMP2 at Thr 514 antibody (1:1000; Abcam, Cambridge, MA) was used to detect and quantify^25^ the expression of p-CRMP2 relative to that of total CRMP2 (clone C4G, 0.2ug-0.4ug/ml; Immuno-Biological Laboratories, Minneapolis, USA).

### Statistics

When three or more intervention groups were compared, inter-group differences were compared with one or two-way ANOVAs, followed by Tukey’s post-hoc tests, as appropriate. When only two groups were compared, two-tailed paired or unpaired Student’s t-tests were performed. A probability of error of *P* < 0.05 was considered statistically significant.

## Electronic supplementary material

Supplementary data files

## Data Availability

The datasets generated during and analyzed during the current study are freely available from the corresponding author on reasonable request.
