# Supplementary material for: Corneal myofibroblasts inhibit regenerating nerves during wound healing
Source: Sci Rep. 2018 Aug 28;8:12945. doi: 10.1038/s41598-018-30964-y (PMC6113331; doi:10.1038/s41598-018-30964-y)
Supplement: Supplementary file 1 — Supplementary data files [file 41598_2018_30964_MOESM1_ESM.pdf]

Supplementary data files for:

Corneal myofibroblasts inhibit regenerating nerves during wound healing

Kye-Im Jeon, Holly B. Hindman, Tracy Bubel, Thurma McDaniel, Margaret DeMagistris, Christine Callan and Krystel R. Huxlin

Supplementary Figures 1-5

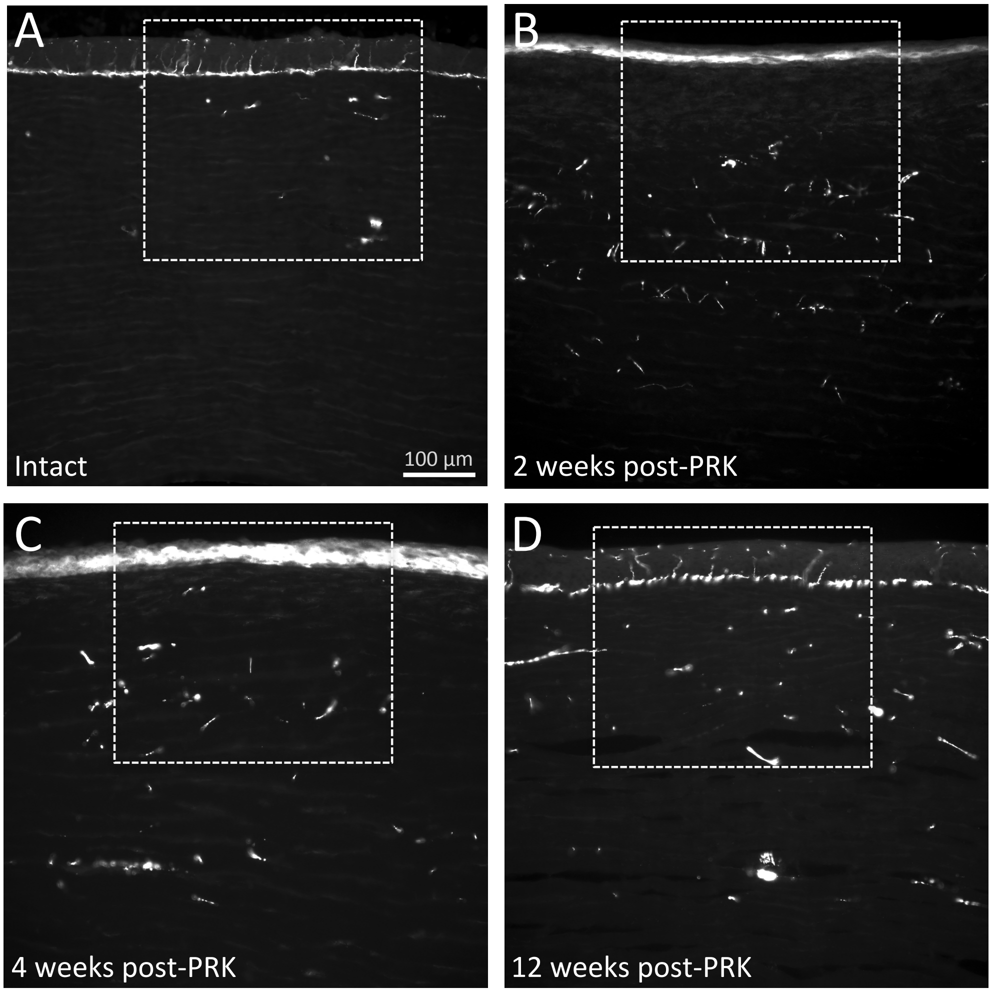

Supplementary Figure 1 (Fig. S1). Monochrome photographs of immuno-stained feline corneas taken using an ORCA-Flash 4.0 LT Digital CMOS camera (Hamamatsu Photonics K.K.). A. Photograph of normal, unoperated central cat cornea illustrating Tuj-1 labeling. The triple labeled version of the dotted rectangular region is shown in Fig. 1A. Thick, fasciculated cords of Tuj-1 positive corneal nerves are evident in the anterior stroma, along with the almost continuous sub-basal nerves right under the epithelium and the thin but rather densely distributed intra-epithelial nerves. **B.** Photograph of the central cornea of a cat 2 weeks after PRK illustrating Tuj-1 staining (the triple labeled version of the dotted rectangular region is shown in Fig. 1B). Note the over-abundance of stromal nerves deeper in the stroma and their absence from the sub-epithelial and epithelial layers, which as seen in Fig. 1B is occupied by α-SMA positive cells. **C**. Photograph of the central cornea of a cat 4 weeks after PRK illustrating Tuj-1 labeling (the triple labeled version of the dotted rectangular region is shown in Fig. 1C). **D.** Photograph of the central cornea in a cat 12 weeks after PRK (the triple labeled version of the dotted rectangular region is shown in Fig. 1D). Thicker trunks of Tuj-1 positive nerves are re-appearing in the stroma, as are intra-epithelial and sub-basal nerves. Scale bar=100µm for A-D.

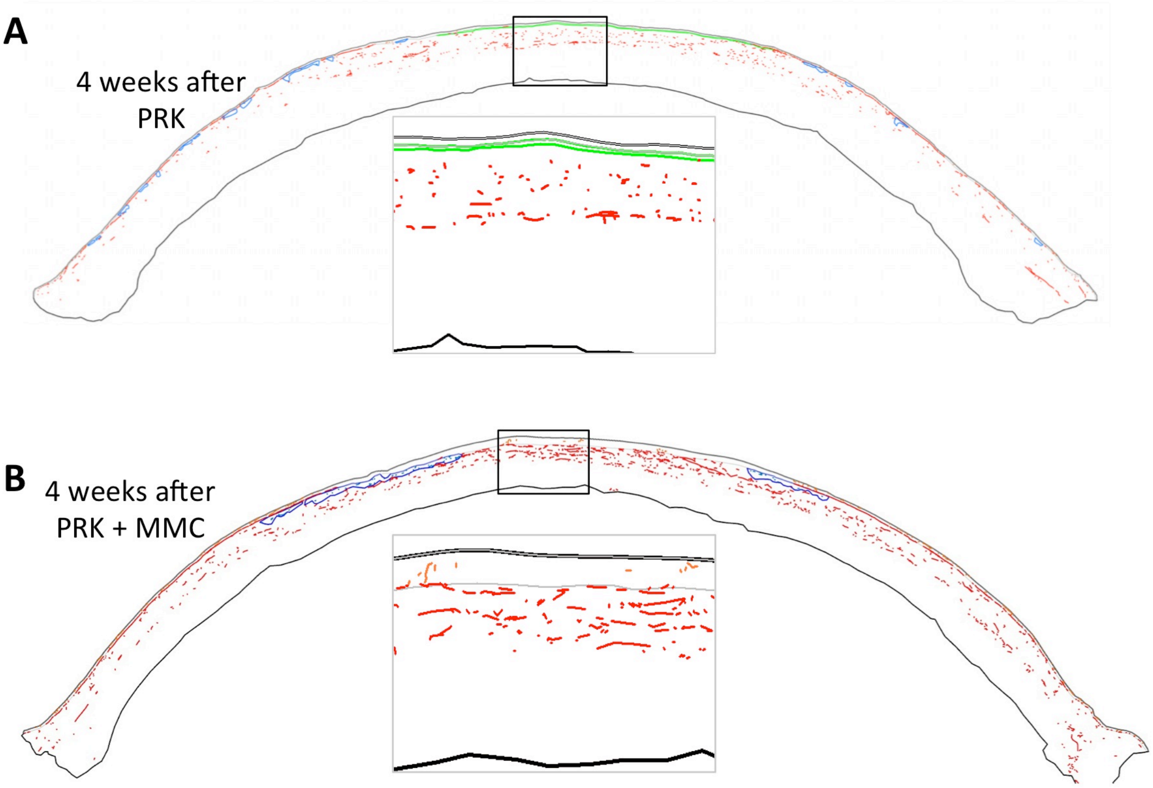

Supplementary Figure 2 (Fig. S2). A. Tracing of an entire cat corneal cross section 4 weeks post-PRK, performed using the Neurolucida software, and illustrating the different color codes used to denote corneal compartments and nerve types shown in Figs. 1 and 2, and which were analyzed quantitatively in Fig. 3. Insets show higher power views of the central region of this cornea. *Corneal zone labels:* thick black outline=outer boundaries of corneal section, thing grey outline=epithelial boundaries, green outlines=boundaries of α-SMA positive zones, blue outlines=boundaries of acellular zones. *Corneal nerve labels*: red=stromal and sub-basal nerves, orange=intra-epithelial nerves, pink=nerves in α-SMA positive zones, blue=nerves in acellular zones. **B.** Tracing of an entire cat corneal cross section 4 weeks post-PRK + MMC, performed using the Neurolucida software, and using color conventions described in A. Note the thinner stromal centrally, the total absence of α-SMA positive zones and the large acellular zones, which are populated by blue-labeled nerves. Unlike the section in A, the central portions of MMC-treated cornea contain sub-basal nerves, and even intra-epithelial nerves in the hyperplastic epithelium. This is in stark contrast with corneas not treated with MMC after PRK (e.g. in A) where there is a total absence of sub-basal and intra-epithelial nerves centrally, in the ablation zone that contains myofbroblasts.

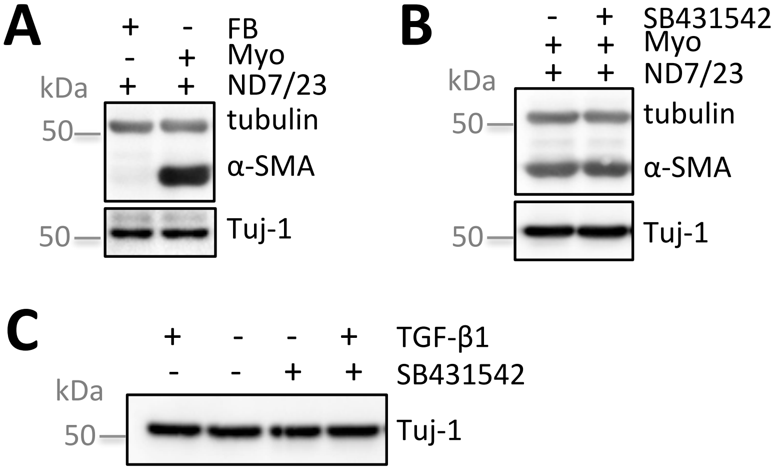

Supplementary Figure 3 (Fig. S3). A. Representative Western blots showing protein levels for α-SMA, Tuj-1, and tubulin obtained from 3 days old ND/FB co-cultures (lane 1) and ND/Myo co-cultures (lane 2). Tubulin was used as a loading control in this experiment. The blots confirmed the presence of Tuj-1 positive cells in both co-culture types, the strong presence of α-SMA positive myofibroblasts in the ND+Myo co-cultures, and their total absence in ND+FB co-cultures. Samples were run on different gels and probed with antibodies against the molecules of interest, as described in the Methods. Full, unedited gels from which the present images are cropped are appended in Supplementary Figure 4. **B.** Representative Western blots showing protein levels for α-SMA and Tuj-1, tubulin obtained from 1 day old ND/Myo co-cultures treated with either DMSO (lane 1) or the TGF-β receptor inhibitor SB431542 (lane 2). Tubulin was used as a loading control in this experiment. As for A, samples were run on different gels and probed with antibodies against the molecules of interest. Full, unedited gels from which the present images are cropped are appended in Supplementary Figure 4. **C.** Representative Western blot showing protein levels for Tuj-1 obtained from 1 day old ND7/23 mono-cultures treated with either TGF-β1 (lane 1), DMSO (lane 2), the TGF-β receptor inhibitor SB431542 (lane 3) or TGF-β1 together with SB431542 (lane 4).

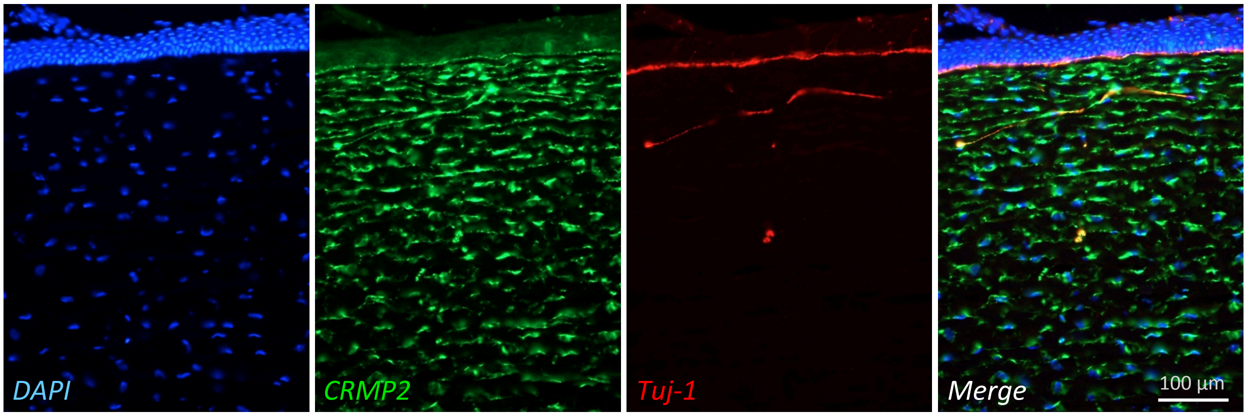

Supplementary Figure 4 (Fig. S4). Feline corneal keratocytes and nerves express CRMP2. Photographs of normal, unoperated cat corneal cross-section reacted for Tuj-1 (red fluorescence), CRMP2 (green fluorescence) and counter-stained with DAPI (blue fluorescence). Note that CRMP2 appears to be strongly expressed by stromal keratocytes in additional to corneal nerves. Cords of corneal nerves are clearly visible as Tuj-1 positive structures in the anterior stroma, the sub-basal layer right under the epithelium and as sparse, thin intra-epithelial nerves. The merged picture on the far right illustrating their double-labeling with CRMP2 antibodies across all compartments of the cornea. Scale bar=100μm for all pictures.

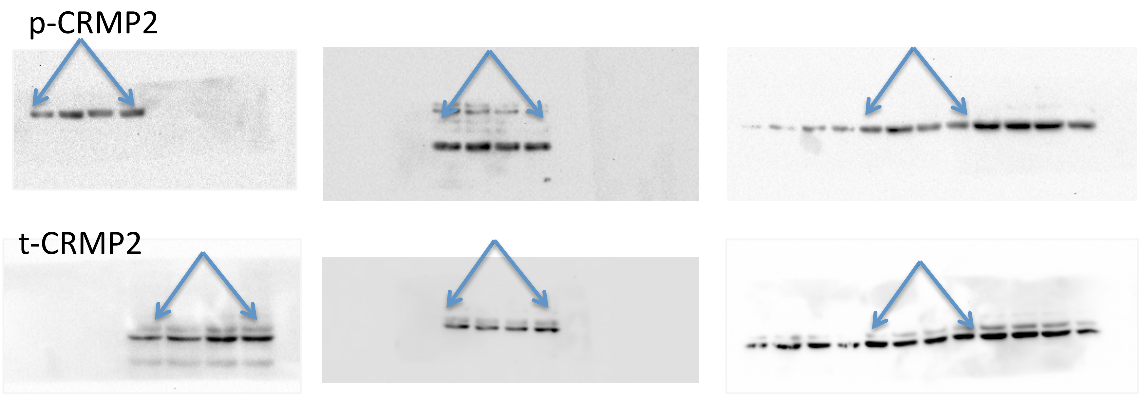

Full unedited gels for Figure 6A, B. Blue arrows indicate lanes analyzed in each gel.

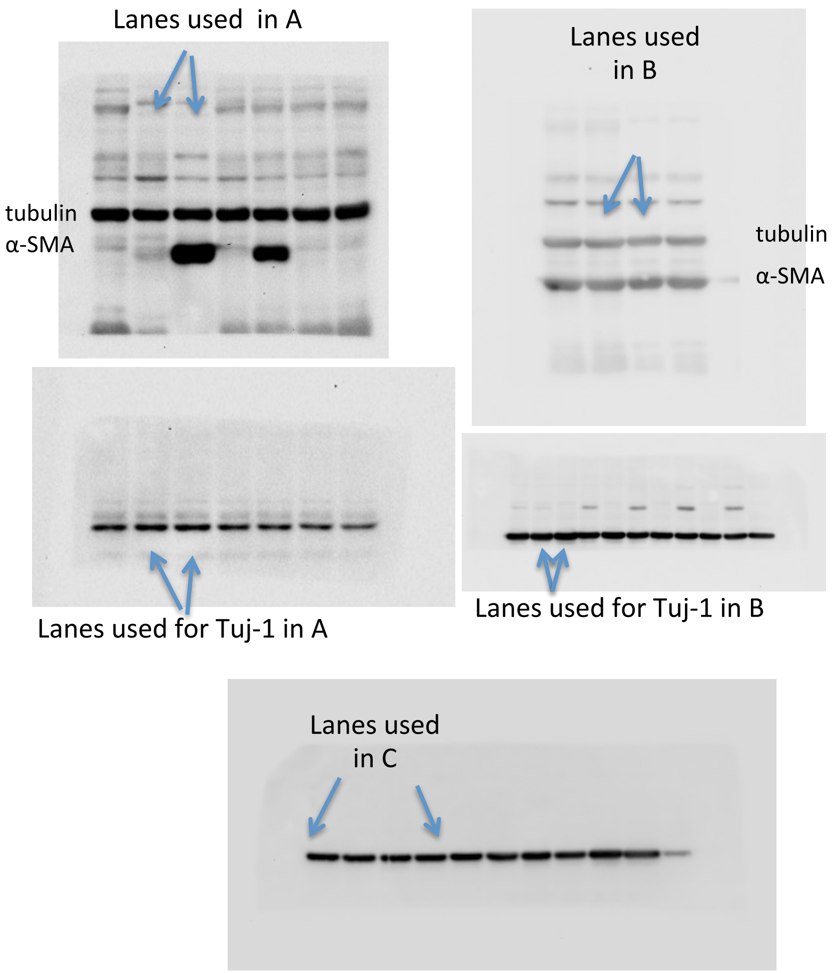

Full unedited gels for Supplementary Figure 3 (Fig. S3)

Supplementary Figure 5 (Fig. S5). Full, unedited gels for Fig. 6 and Fig. S3.
